# Meta-analysis of studies on the impact of mobility disability simulation programs on attitudes toward people with disabilities and environmental in/accessibility

**DOI:** 10.1371/journal.pone.0269357

**Published:** 2022-06-10

**Authors:** Gloria Yuet Kwan Ma, Winnie W. S. Mak

**Affiliations:** Department of Psychology, The Chinese University of Hong Kong, Hong Kong, China; Xiamen University - Malaysia Campus: Xiamen University - Malaysia, MALAYSIA

## Abstract

The reported equivocal evidence of the effectiveness of disability simulation programs in reducing ableist attitudes toward people with disabilities has led to a persistent debate about the suggested discontinuation of such simulation programs to avoid further reinforcement of ableism. The present research conducted a meta-analysis on 12 empirical studies evaluating the impact of mobility disability simulation programs on attitudes toward people with disabilities and environmental accessibility to better inform future research and practice. A citation search using keywords related to “disability” and “simulation” in the title and/or abstract in 11 major online databases (i.e., Cochrane, EBSCOhost, EMBASE, Google Scholar, IEEE Xplore, JSTOR, LearnTechLib, ProQuest, PsycINFO, Scopus, and Web of Science) was conducted to retrieve relevant empirical articles that are published within the earliest dates of each database and June 2021 for the meta-analysis. Meta-analysis using a random effects model revealed that participation in the simulation programs resulted in large effect sizes in increasing fear and anxiety [Cohen’s *d* = -1.51, 95% *CI* (-2.98, -.05), *n* = 2] but small effect sizes in improving conceptions of social inclusion at postsimulation [Cohen’s *d* = .24, 95% *CI* (.01, .47), *n* = 5] while reducing stereotypes toward people with disabilities at follow-up [Cohen’s *d* = .57, 95% *CI* (.10, 1.03), *n* = 3]. Inconclusive changes in the behavioral tendency of inclusion-promoting actions and stereotypes at postsimulation were found. The three exploratory moderators (i.e., the program duration, the presence of facilitators with disabilities, and the debriefing arrangement) were not statistically significantly associated with between-subgroup differences in the program’s effectiveness in reducing stereotypes toward people with disabilities. The findings informed a series of recommended reforms in the program message framing, formats of the simulation, scope and referents of outcome measures, incorporation of environmental perspectives and behavioral measures, and methodological quality of the program evaluation study.

## Introduction

Ableism is the stigmatizing preference for normatively “*healthy*” or “*abled*” individuals that is against people with disabilities [1, 2]. Due to ableism, people without disabilities are unable to recognize with the stigmatizing experiences of environmental inaccessibility encountered by people with disabilities [3–5]. For example, such an ableist mindset would hinder the rapport between college administrative staff, teaching staff, and students with disabilities. It would in turn impair the establishment of inclusive campus.

Disability simulation programs have been extensively adopted to dismantle ableist attitudes toward people with disabilities in educational context [3, 6–9]. The programs are particularly popular in the professional training of medical and nursing practitioners, as well as engineers, designers, and social workers given its flexibility in the duration and content [10–15]. Depending on the resource availability and time constraints in practice, it might not always be feasible to conduct multiple types of disability simulation simultaneously. Simulating mobility disability using assistive devices such as wheelchairs and crutches is a very popular approach in disability simulation programs in many educational settings. The present meta-analytic review focused on the simulation of mobility disability.

The present meta-analytic review followed the Tripartite Model of Attitudes [16–18] and incorporated environmental perspectives by taking negotiations with environmental in/accessibility into consideration [19, 20] to better inform future research and practice [16]. Based on the Tripartite Model of Attitudes [19, 21], a multidimensional approach to ableist attitudes has been advocated by conceptualizing attitudes into three different domains, namely stereotypes (cognition), prejudices (emotion), and discriminations (behavior), for more comprehensive analysis. Kim and colleagues [18] found medium to large intercorrelations of ableist cognition, affect, behaviors at r = 0.27 to 0.59. Empirical assessment of cognitive, emotional, and behavioral responses of people simulating mobility disability toward responses to negotiations with environmental in/accessibility is in paucity.

### Mechanisms of disability simulation programs in addressing ableist stereotypes, emotions, and discriminations

During typical programs of mobility disability simulation, the participants (who often have no self-reported disability) simulate certain mobility disability by various physical means such as using wheelchairs or crutches to travel around and/or perform certain tasks. Then, the participants are readily exposed to different scenarios of environmental in/accessibility in the presence of the simulated mobility disability [6, 22, 23]. Negotiations with environmental in/accessibility happen when there is encounter of difficulty along with threats to our dignity in wayfinding, entering, and/or circulating in certain environments due to inaccessibility [24]. The literature suggested that it is this embodied process of coping with these negotiations that might address ableist attitudes toward people with disabilities and environmental in/accessibility [25]. However, equivocal evidence of the relevant disability simulation programs is consistently reported in the literature [6, 8, 11, 14, 20, 26, 27].

Various emotional reactions, particularly anger, fear, frustration, helplessness, and embarrassment, in response to the direct experiences of denied choices and opportunities due to first-hand experiences of environmental inaccessibility during simulation programs were commonly reported by the participants [8, 14]. These immediate emotional responses by the participants highly echo those negative emotions reported by people with mobility disability and their affiliates (e.g., family members, friends, caregivers) in face of environmental barriers in everyday life [25, 28].

Although some past studies interpreted these immediate emotional outcomes as undesirable consequences of these simulation programs [8, 14], it might be turn out to be associated with the empathetic understanding of environmental barriers to challenge ableism [29, 30]. It might promote the awareness of the stigmatizing nature and detrimental impact of environmental inaccessibility [8, 14]. The enhanced empathic understanding of stigmatizing experiences might also drive the participants to attend to the underlying injustice regarding the environmental inaccessibility [31], and to identify with inclusion-affirming advocacy groups or allies advocating for a barrier-free and inclusive society.

Furthermore, participants must actively brainstorm and try out different solutions to cope with any embodied negotiations with environmental inaccessibility during the simulation programs. The real-time dynamic feedback of the interactions received from the surroundings and other people, e.g., pedestrians, shop staff, and/or their companions, during simulation programs could coalesce to offer participants concrete insights into how to interact with people with mobility disability in a mutually more respectful and empathetic manner [9, 31, 32].

Participants might realize that some of their preexisting stereotypes and behaviors toward people with disabilities were stigmatizing or invalid based on their embodied experiences during the simulation programs. For instance, they might become more conscious that ableist behaviors such as uninvited help, civil inattention, or unfriendly gaze could be patronizing or even offensive even if they apparently look “harmless” or “with-good-intention” [25, 33]. This type of experience might remind them to avoid these discriminatory behaviors in their social encounters with people with disabilities in their everyday life. This experience might enhance the behavioral efficacy to modify or even eliminate those invalid stereotypes and to construct and adopt less-stigmatizing perceptions [3, 7, 8, 34, 35]. It would help bring about actual changes to challenge ableism.

On the other hand, undesired influences of the relevant disability simulation programs have been reported in the literature [6, 8, 11, 14, 20, 26, 27]. Besides the above-mentioned emotional responses such as fear, frustration, helplessness, and embarrassment, pity and sympathy could be induced simultaneously. The salience of environmental barriers experienced during the simulation programs might constitute overwhelming environmental sources that confirm and sustain the preexisting shared reality of the culturally justified “abnormality” and “minority” status of people with disabilities [4, 8, 36–38]. It would in turn substantiate the stereotypical and deficit orientation of disability. They might readily establish an ableist causation that those negotiation experiences must be caused by the identity of having mobility disability. Such simulation experiences might reinforce the stereotype that living with mobility disability is tragic and helpless and that individuals with mobility disability are sufferers [14, 36]. Participants who experience these emotional responses might tend to further avoid people with mobility disability and/or accessibility issues.

### Moderators of the simulation program’s effectiveness

Flower and colleagues [6] found that the meta-analyzed effect size for mixed types of disability simulations of less than 30 minutes (Cohen’s *d* = .54) was relatively largest among thirteen studies on mixed types of disability simulations for reducing negative attitudes toward people with disabilities, followed by those of an hour or more (Cohen’s *d* = .35), then those within 30–60 minutes (Cohen’s *d* = .03). Jeon [39] reported a very large meta-analyzed effect sizes for mixed types of disability simulations of less than 800 minutes (median Cohen’s *d* = 1.25) but almost medium effect size for mixed types of disability simulations of 800 minutes and more (median Cohen’s *d* = .45). There seems to be a trend of inverse associations of the program duration and the magnitude of the meta-analyzed effect sizes. Herbert [13] suggested that disability adjustment might vary across contexts and over time. Desired and authentic embodied experiences, stereotype reevaluation, and attitudinal changes might not be achievable within only a short period of time because adjustment to disability takes time [13]. On the other hand, unfamiliar and overwhelmingly stigmatizing experiences might accumulate as the program continues. Different durations of simulation programs may create opposite effects.

Program facilitation by people with disabilities is a kind of direct contact approach that might bring about positive effects on reducing ableist attitudes toward people with disabilities more strongly and reliably. Empirical evidence showed significant and positive associations of direct contact with people with disabilities and more inclusive attitudes toward people with disabilities [5, 17, 40–43]. People with disabilities could directly reify more concrete and nonableist beliefs and behavioral examples [40, 44], e.g., by raising critical consciousness of ableist microaggressions [33, 45], educating the public through interpersonal dialogue in daily life, reducing the use of ableist language in daily life [38, 46–51]. They could help decipher the underlying ableist societal systems and functioning during the simulation programs. By explicitly incorporating the first-hand account of living experiences with disability throughout the simulation programs, they could help demystify how they cope with disability and negotiations with environmental in/accessibility in everyday life, e.g., through adapted behavior patterns, planning ahead, educating the public, claiming, and downplaying [28, 52].

Participants of the simulation programs might then possess at least three essential perspectives and reactions toward people with mobility disability and negotiations with environmental in/accessibility. The three perspectives include 1) “observers” (participants’ preexisting perspectives), 2) “novice users” (participants’ perspectives of temporarily in the state of simulating mobility disability), and 3) “expert users” (people with disabilities’ perspectives), to establish empathetic understanding and desired reappraisal of preexisting stereotypes. Moreover, interpersonal interactions between the facilitators with disability and the participants during the process of simulation programs might drive participants’ affiliative motivation to align with the ableism-reducing beliefs, emotions, and behaviors conveyed by the facilitators with disability to achieve a better sense of shared reality toward social inclusion [53].

Past studies have emphasized that debriefing is essential for addressing any elicited feelings and thoughts, confusion, questions, and concerns immediately upon the completion of the simulation programs to maximize adherence to the program objectives and desired reduction in ableist attitudes [3, 8, 9]. It could allow a period for participants to reorganize and make sense of any feelings and thoughts evoked during the simulation programs or to discuss the simulation experiences with the program facilitators and/or other participants. It is required for the effects of the embodied experiences to be assimilated into participants’ preexisting cognitions, emotions, and behaviors to effectively bring about the attitude’s reevaluation and desired changes [54].

### Research hypotheses in the present meta-analysis

Based on the equivocal program outcomes discussed in the literature review above, an exploratory meta-analytic review (i.e., instead of specifying hypothesized directions of program effects) on the impact of the simulation programs on attitudes toward people with disabilities and environmental in/accessibility was conducted. Specifically, it was hypothesized in the present meta-analysis that the empirical studies on mobility disability simulation programs would show any impact on the following five domains at a) postsimulation, and b) at follow-up, respectively: (1) stereotypes toward people with mobility disability; (2) discrimination against people with mobility disability; (3) conception of environmental in/accessibility; (4) behavioral tendency of inclusion-promoting actions, and (5) emotions.

Exploratory meta-analytic review on the moderating effects of the three study-level moderators were also conducted. It was hypothesized that simulation program design features, namely, (1) the program duration; (2) the presence of facilitators with disability; and (3) the debriefing arrangement, would moderate the program effectiveness, respectively.

## Method

The meta-analysis was conducted in accordance with the PRISMA checklist [55] (S1 Table). The meta-analysis was not preregistered, and review protocol was unavailable.

### Identification of studies

A citation search in eleven major online databases was conducted to retrieve empirical studies evaluating the impact of mobility disability simulation programs published between the earliest dates of each database and June 2021: Cochrane, EBSCOhost, EMBASE, Google Scholar, IEEE Xplore, JSTOR, LearnTechLib, ProQuest, PsycINFO, Scopus, and Web of Science. The citation search was conducted using keywords related to “disability” and “simulation” in the title and/or abstract. Specifically, the keywords included “disability”, “disabilities”, “disabled”, “handicap”, “handicaps”, “handicapped”, “handicapping”, “impairment”, “impairments”, “impaired”, “experiential”, “simulation”, “simulations”, “simulated”, “simulating”, and “awareness”. The word “mobility” was not included in the keyword search in order to cover a wider scope of relevant citations, because in general “mobility disability simulation programs” are commonly called “disability simulation programs” in the literature. The electronic search strategy for Web of Science is presented in S1 File. A manual search within the bibliography of studies included in the present meta-analysis and that of the two published meta-analysis of disability simulations [6, 39] and two systematic reviews on disability awareness programs [26, 56] was performed to identify further studies that might be missed in the database search. The search was completed in June 2021.

### Inclusion and exclusion criteria

**Fig 1:**
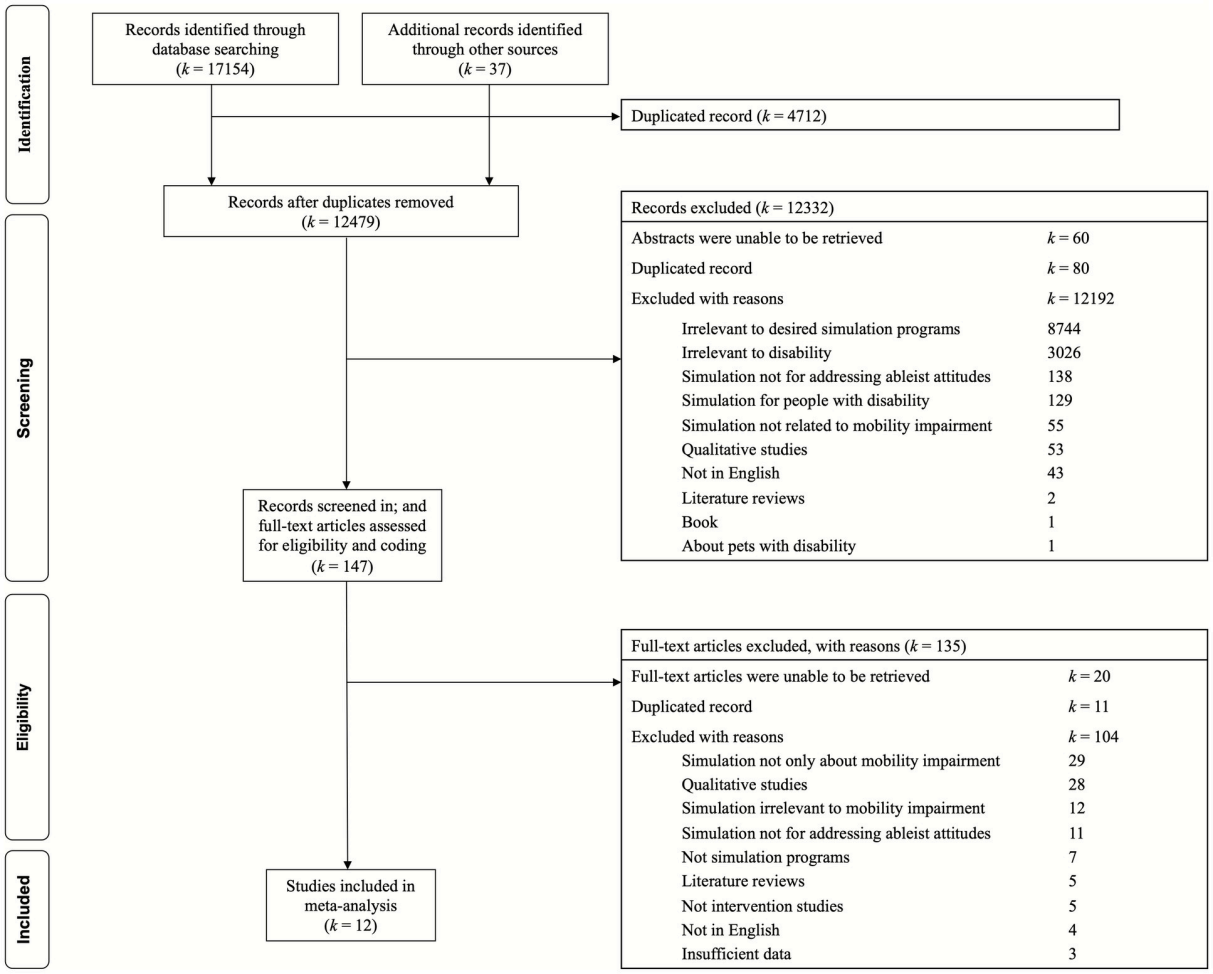
PRISMA study selection flow chart.

The titles and abstracts of the studies identified from the citation search were initially screened based on the following inclusion and exclusion criteria. The PRISMA flowchart [55] was used to record details of the different phases of inclusion/exclusion and coding of articles in the meta-analysis (Fig 1).

Studies were included if they: (1) were interventions evaluating the impact of mobility disability simulation programs on ableist attitudes (including stereotypes, prejudices, discriminations) with reported statistical data for effect size calculations; and (2) had at least one of the five hypothesized outcome variables. No restrictions on the publication type (e.g., journal articles, unpublished dissertations), sample size, sample type (as long as they did not have any self-reported disability), deliverers, simulation format (e.g., using wheelchairs or crutches), simulation duration, or follow-up period length were applied to the screening.

Studies were excluded if they: (1) were mobility disability simulations but did not aim at influencing ableist stereotypes, emotions, behaviors, and/or general attitudes; (2) involved simulations of any type of disability (e.g., visual or hearing) other than mobility disability; (3) involved simulations of any type of disability simultaneously with mobility disability; (4) employed nonembodied formats of simulation, such as vicarious observations through videotapes; (5) were qualitative in nature (e.g., narrative reviews and commentaries); (6) focused on participants with disability; or (7) were not written in English.

### Data extraction

Full texts of the studies retained from the initial screening were inspected, and the following data were coded. Two coders independently coded the included articles, and the intercoder agreement was 94.8%.

The coded data included: sample characteristics (mean age, gender composition, sample type, country of origin, any reported disability); program characteristics (e.g., program content, formats of the simulation of mobility disability, program duration, involvement of any facilitators with disability, debriefing arrangement); outcome assessment (e.g., operationalization of the outcomes and name and reliability of the instruments); statistical data for effect size calculations (e.g., mean scores and the corresponding standard deviations, *p*-values of mean changes, sample sizes, directions of change); and the methodological quality. If the reported statistical data was insufficient for effect size calculations, attempts to request the required data from the study authors were made.

Methodological quality of the included studies was assessed based on both the NIH Quality Assessment Tool for Before-After (Pre-Post) Studies with No Control Group [57] and the NIH Quality Assessment Tool for Controlled Intervention Studies [58]. The adapted assessment criteria included: sampling method, random assignment, allocation concealment, blinding, baseline differences, drop-out, adherence, response rate, instruments’ reliability, power calculation, and intention-to-treat analysis (S2 Table). Fulfillment of these assessment criteria indicated a study with better methodological quality. Lower methodological quality suggested lower internal validity of the results, inferring greater risk of bias in the findings.

### Data analysis

Statistical analyses were all conducted in Comprehensive Meta-Analysis Version 3.0 [59]. The standardized mean difference (Cohen’s *d*) was the adopted effect size statistic in the present meta-analysis, where *d* = .2, .5, and .8 indicated small, medium, and large effect size, respectively [60]. The pre- and postsimulation assessments were defined as the assessments conducted before and immediately upon the completion of the simulation programs, respectively. Follow-up assessments were defined as those assessments conducted at certain period of time following the postassessments.

A random effects model was adopted to pool the individual effect sizes due to the anticipated variety of program designs, sample types, and the instruments of the outcome measures. This approach assumed that the true mean scores of the effect sizes of the hypothesized changes varied across studies and that the true effects were normally distributed. If there was more than one independent sample within the same study, effect size calculation was conducted separately for each independent sample. If there were multiple measures of the same outcome or multiple comparison groups with dependent samples, effect sizes of these multiple measures or groups were averaged to generate a single effect size. If there was more than one control group compared with the simulation (intervention) group, then the statistical data of the control groups was first averaged, and this averaged control group data was employed to proceed. These measures avoided the violation of the assumption of study independence.

The statistical significance of the effect size estimates was determined by their 95% confidence intervals (*CI*), with values cutting across zero indicating statistical nonsignificance. Positive effect size estimates indicated reduced stereotypes toward and discriminations against people with mobility disability, improved conception of environmental in/accessibility, enhanced behavioral tendency of inclusion-promoting action, and an increase in positive emotions after participation in the simulation, respectively. Negative effect sizes indicated increased stereotypes toward and discriminations against people with mobility disability, more ableist conceptions of environmental in/accessibility, reduced behavioral tendency of inclusion-promoting actions, and an increase in negative emotions after the simulation, respectively. Directions of effects were dependent on each individual study.

Heterogeneity of the pooled effect size estimates was examined by the Cochran’s *Q* statistic, which assessed whether the observed heterogeneity in the effect size estimates was compatible with chance alone [61]. Statistically significant heterogeneity among the effect sizes was indicated by Cochran’s *Q* at *p* < .05. Heterogeneity was quantified by the *I*^2^ statistic [61], which measured the proportion of the observed variance across studies that reflected heterogeneity in true effect sizes rather than chance. The 95% *CI*s of the *I*^2^ statistic were computed using the formula in Borenstein and colleagues [62], with values cutting across zero indicating statistical nonsignificance. Borenstein and colleagues [62] recommended that *I*^2^ values of 25%, 50%, and 75% suggested low, medium, and high heterogeneity, respectively. Sedgwick [63] suggested that a significant Cochran’s *Q* along with the *I*^2^ value approaching 50% or higher indicated the presence of heterogeneity.

Subgroup analyses were conducted using a mixed effects model [62] to test the hypothesized three categorical study-level moderators, namely, the simulation duration (categorized into an hour or less; more than an hour but within one day; and 1–2 days), facilitation by people with disabilities (categorized using a binary code of yes or no), and debriefing arrangement (categorized into interactive debriefing, one-way written account only, simple debriefing, and no debriefing), respectively. Interactive debriefing was defined as an interactive period held immediately after the simulation session, such as mutual sharing of emotions and thoughts, spontaneous feedback giving, and question-and-answer sessions among participants and facilitators. A one-way written account was defined as a written account of any free responses to the simulation experience that were submitted to the program organizers without receiving any feedback afterward. Simple debriefing was defined as one-way explanation of the research purposes and details to the participants at the end of the simulation by the facilitators or program organizers without an interactive period as defined above.

The random effects model was used to pool individual study effect size within each subgroup, and the statistical significance of the pooled effect sizes was determined by the 95% *CI*s. Heterogeneity in effect sizes within each subgroup was assessed by the Cochran’s *Q* and the *I*^2^ statistic. The fixed effects model was used to pool the averaged effect size across the subgroups. The Cochran’s *Q*_*Between*_ (*Q*_*B*_) was used to test for statistically significant variations (rather than random errors and chance) in the pooled effect sizes across the subgroups of each categorical study-level moderator. The alpha level for determining statistical significance of the Cochran’s *Q*_*B*_ was Bonferroni-corrected at 0.05 / 4 = 0.0125 [62] for three hypothesized and one posterior subgroup analyses. The *I*^2^ statistic for variations across subgroups were computed using the formula in Deeks and Higgins [64] with the 95% *CI*s cutting across zero indicating statistical nonsignificance. This between-subgroup *I*^2^ statistic measured the proportion of total variation in subgroups’ effect size estimates that was due to genuine variation across the subgroups rather than to sampling error [64]. Higgins and Green [65] recommended that, as a convention, at least ten independent studies (n = 10) are required for subgroup analysis to be conducted.

Publication bias was first assessed by visual inspection of the funnel plots [66]. Funnel plot is a scatter plot of the effect size estimates (standardized difference in means) from individual studies (i.e., the x-axis) against the standard error of each corresponding effect size estimate (i.e., the y-axis). The precision of effect size estimates from studies would increase as the sample size of the corresponding study increases [65]. The effect size estimates from studies with smaller sample sizes would scatter relatively more widely around the bottom of the plot while the effect size estimates from studies with larger sample sizes would scatter relatively more narrowly around the top part of the plot. The plot would resemble an inverted and symmetrical funnel in the absence of biased results. Uneven distribution of studies within the bottom right area of the funnel indicated that studies with positive results might tend to be published and thus retrieved for inclusion in the meta-analysis.

To further quantify the amount of bias captured by the funnel plot, the Begg and Mazumdar’s test [67] and the Egger’s test [68] were conducted. In the Begg and Mazumdar’s test [67] reported the rank correlations (Kendall’s tau) between the standardized effect size estimates and the standard errors of the corresponding standardized effect size estimates [62, 65]. Two-tailed statistical significance test on the reported Kendall’s tau was conducted. A statistically significant Kendall’s tau (*p* < .05) would suggest the presence of bias. For the Egger’s test [68], linear regression of the standardized effect size estimates on the inverse of the standard error of the corresponding standardized effect size estimates was conducted [62, 65]. Two-tailed statistical significance test on the intercept in this linear regression was conducted. A statistically significant intercepts (*p* < .05) would suggest the presence of bias.

Duval and Tweedie’s [69] trim-and-fill method was also conducted. It was an iterative process of removing (i.e., trimming) the smaller studies causing funnel plot asymmetry, re-computing the overall effect size until the funnel becomes symmetrical, then replacing (i.e., filling) the omitted studies and their mirror images around the center of funnel center to correct the variance [62, 65]. This trim-and-fill method estimated effect sizes adjusted for the possible publication bias by performing a meta-analysis including the filled studies [65]. It estimated the number of studies that were missing for symmetrical funnel plots, with a greater number of required studies indicating larger extent of publication bias [62, 65].

Overall, Sterne and Egger [66] recommended that the conventional minimum number of independent studies for valid publication bias assessments to be conducted with satisfactory power was ten independent studies.

## Results

### Flow of study identification, inclusion, and exclusion

A total of 17191 citations were identified from the citation search. Then, 4712 duplicated citations were removed, leaving 12479 citations for the initial screening of titles and abstracts. Based on the inclusion and exclusion criteria for the initial screening, a total of 12332 citations were excluded for various reasons, while 147 citations were retained for full-text examination for coding. Among the 12332 excluded citations, 80 citations were duplicates, and the abstracts of 60 citations were nonretrievable. Upon examination of those 147 full texts, 135 articles were further excluded with reasons. Twelve out of these 147 articles were retained in the final meta-analysis. The flow of study selection and reasons for study inclusion and exclusion are listed in detail in Fig 1.

### Characteristics and methodological quality of the included studies

The main characteristics of the sample, simulation design, and relevant outcomes of each of the twelve articles retained in the present meta-analysis are presented in the S3 Table, and a summary is presented in the S4 Table. The reported information in the included studies was insufficient for comprehensively assessing methodological quality. Criteria of the methodological quality assessment are presented in the S2 Table, and the assessment results are presented in the S5 Table.

The included studies framed their programs as “disability simulation” (k = 7), “disability awareness program” (k = 2), “Paralympic School Day” (k = 2), or “simulated disability sensitivity training” (k = 1). The studies employed either a before-and-after design (k = 5 for uncontrolled studies without follow-up; k = 2 for having nonrandomized controlled groups without follow-up; and k = 1 for having nonrandomized groups with follow-up) or randomized controlled trials (k = 3 without follow-up and k = 1 with follow-up).

Approximately 1076 participants were involved (i.e., only those relevant comparison groups were counted), among whom approximately 107 participated in their corresponding follow-up assessments. Participants were mainly undergraduates and postgraduates (k = 7). Most of the studies employed wheelchair-use as the only (k = 7) or one of the formats (k = 3) of simulating mobility disability. The most commonly used instrument for the outcome assessment was the Attitudes Toward Disabled Persons (k = 5; Forms A/B/O) Scales [70]. The available outcome variables for pre-post comparisons only included the stereotypes toward people with disabilities (k = 4 using the referent of “people with disabilities”; k = 4 using the referent “disabled persons”; k = 1 using the referent of “people with physical disability”), the conception of social inclusion (k = 3), behavioral tendency of inclusion-promoting actions (k = 5), and the overall emotional changes (k = 2). Therefore, hypotheses regarding the pre-post comparisons on the discrimination against people with mobility disability and the conception of environmental in/accessibility were unable to be further examined. However, the outcome of the change in the conception of social inclusion was retained for further meta-analysis.

The available outcome variables for pre-follow-up comparisons included only the stereotypes toward people with disabilities (k = 2 using the referent of “disabled persons”). Reported statistical data on the hypothesized outcomes of discriminations against people with mobility disability and the conception of environmental in/accessibility were unavailable. As a result, meta-analysis on the outcomes of discrimination against people with mobility disability, conception of environmental in/accessibility, behavioral tendency of inclusion-promoting actions, and emotions, at follow-up were unable to be further examined.

### Meta-analysis on mobility disability simulation program effectiveness

**Fig 2:**
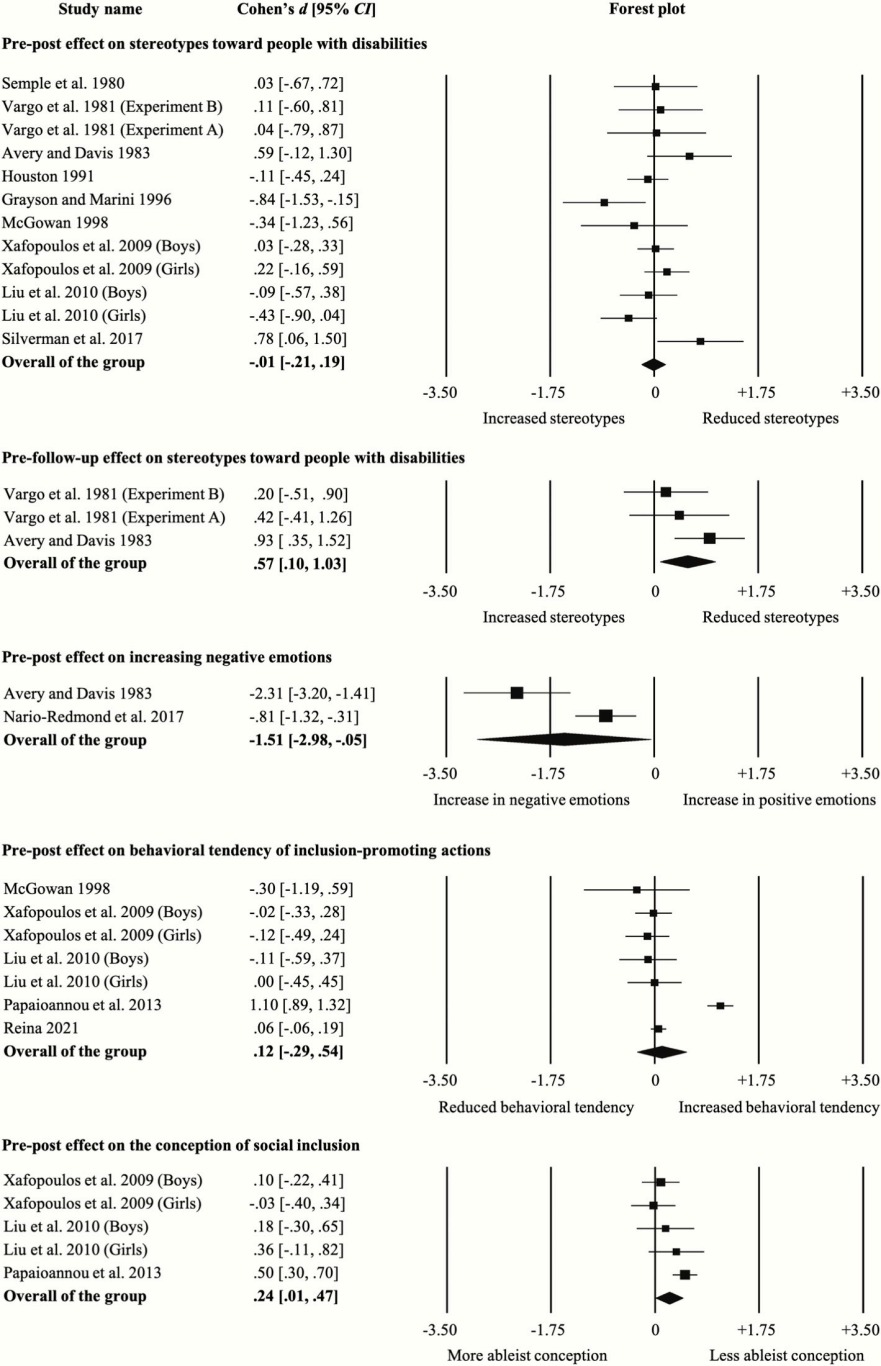
Forest plot.

Summary effect sizes are presented in Table 1. Effect sizes are presented in the forest plots (Fig 2) in ascending order of publication year for each outcome at each comparison time-point, i.e., pre-post and pre-follow-up, respectively.

**Table 1:** Effect sizes of the studies included for meta-analysis.

*k*	Cohen’s *d* [95% *CI*]	*SE*	Cochran’s *Q*	*I*^*2*^ [95% *CI*]	*k*	Cohen’s *d* [95% *CI*]	*SE*	Cochran’s *Q*	*I*^*2*^ [95% *CI*]
**Pre-post effect on increasing stereotypes toward people with disabilities**					**Pre-follow-up effect on reducing stereotypes toward people with disabilities**				
9 articles (12 studies)	-.01 [-.21, .19]	.10	18.52	40.61 [0, 69.89]	2 articles (3 studies)	.57 [.10, 1.03]	.24	2.67	24.99 [0, 97.48]
**Pre-post effect on increasing behavioral tendency of inclusion-promoting actions**					**Pre-follow-up effect on behavioral tendency of inclusion-promoting actions**				
5 articles (7 studies)	.12 [-.29, .54]	.21	81.15***	92.61 [87.29, 95.70]	--	--	--	--	--
**Pre-post effect on improving the conception of social inclusion**					**Pre-follow-up effect on the conception of social inclusion**				
3 articles (5 studies)	.24 [.01, .47]	.12	8.90	55.07 [0, 83.41]	--	--	--	--	--
**Pre-post effect on increasing negative emotions**					**Pre-follow-up effect on the overall emotional changes**				
2 articles (2 studies)	-1.51 [-2.98, -.05]	.75	8.11**	87.67 [52.18, 96.82]	--	--	--	--	--

Positive effect size estimates indicated reduced stereotypes toward people with disabilities, enhanced behavioral tendency of inclusion-promoting actions, improved conception of social inclusion, and increase in positive emotions after participation in the simulation, respectively. Negative effect sizes indicated increased stereotypes toward people with disabilities, reduced behavioral tendency of inclusion-promoting actions, more ableist conception of social inclusion, and increase in negative emotions after the simulation, respectively.

** *p* < .01,

*** *p* < .001.

#### Pre-post simulation comparisons

Meta-analysis using random effects models showed a very small and statistically nonsignificant effect size in increasing stereotypes toward people with disabilities (*d* = -.01, *SE* = .10, 95% *CI* [-.21, .19]; *I*^2^ = 40.61%, 95% *CI* [0, 69.89], *k* = 9, *n* = 12). A small and statistically significant effect size in improving the conception of social inclusion was found (*d* = .24, *SE* = .12, 95% *CI* [.01, .47]; *I*^2^ = 55.07%, 95% *CI* [0, 83.41], *k* = 3, *n* = 5). Effect size in increasing negative emotions was very large and statistically significant (*d* = -1.51, *SE* = .75, 95% *CI* [-2.98, -.05]; *I*^2^ = 87.67%, 95% *CI* [52.18, 96.82], *k* = 2, *n* = 2). A small and statistically nonsignificant effect size in enhancing behavioral tendency of inclusion-promoting actions was found (*d* = .12, *SE* = .21, 95% *CI* [-.29, .54]; *I*^2^ = 92.61%, 95% *CI* [87.29, 95.70], *k* = 5, *n* = 7).

#### Pre- to follow-up comparisons

Meta-analysis using a random effects model showed a large and statistically significant effect size in reducing stereotypes toward people with disabilities from presimulation to follow-up (*d* = .57, *SE* = .24, 95% *CI* [.10, 1.03]; *I*^2^ = 24.99%, 95% *CI* [0, 97.48], *k* = 2, *n* = 3).

#### At both pre-post and pre-follow-up comparisons

The values of the *I*^2^ statistics of most of the outcomes suggested moderate to high (*I*^2^ = 55.07–92.61) heterogeneity, except that the values of the pre-post (*I*^2^ = 40.61) and pre-follow-up (*I*^2^ = 24.99) comparisons of the stereotypes toward people with disabilities were relatively low.

#### Subgroup analysis of moderator testing

Subgroup analysis was conducted only for the pre-post comparisons of stereotypes toward people with disabilities, as this was the only outcome comparison that fulfilled the convention of minimum n = 10. The results of the subgroup analyses are presented in detail in Table 2.

**Table 2:** Results of subgroup analyses on pre-post effects on stereotypes toward people with disabilities.

		*k*	Cohen’s *d* [95% *CI*]	*SE*	*I*^*2*^ [95% *CI*]	*Q*_*B*_	*I* ^2^_between_
**Simulation duration**	*An hour or less*	2 articles (2 studies)	-.65 [-1.21, -.08]	.29	.00 [0, 0]	4.94	59.53
	*More than an hour but within one day*	3 articles (5 studies)	.02 [-.19, .23]	.11	45.17 [0, 79.89]		
	*1–2 days*	2 articles (3 studies)	.06 [-.38, .50]	.23	.00 [0, 0]		
**Facilitation by people with disabilities**	*Yes*	2 articles (4 studies)	-.05 [-.37, .27]	.16	34.79 [0, 77.21]	.11	.00
	*No*	7 articles (8 studies)	.02 [-.27, .31]	.15	49.67 [0, 77.53]		
**Debriefing arrangement**	*Interactive debriefing*	1 article (1 study)	-.34 [-1.31, .63]	.49	.00 [0, 0]	4.43	32.33
	*One-way written account only*	2 articles (3 studies)	.06 [-.42, .54]	.24	.00 [0, 0]		
	*Simple debriefing*	1 article (1 study)	.78 [-.03, 1.59]	.41	.00 [0, 0]		
	*No debriefing*	5 articles (7 studies)	-.07 [-.29, .15]	.11	53.81 [0, 80.27]		
**Referents of the measures of stereotypes toward people with disabilities**	*“People with disabilities”*	4 articles (6 studies)	.01 [-.20, .22]	.11	47.53 [0, 79.20]	5.17	61.28
	*“People with physical disability”*	1 article (1 article)	-.84 [-1.60, -.09]	.39	.00 [0, 0]		
	*“Disabled persons”*	4 articles (5 studies)	.12 [-.25, .48]	.19	.00 [0, 0]		

Positive and negative effect size estimates indicated reduction and increase in stereotypes toward people with disabilities, respectively.

Simulation duration was not statistically significantly associated with between-subgroup differences in the program’s effectiveness in reducing stereotypes toward people with disabilities [*Q*_*B*_(2) = 4.94, *p* = .09; *I*^2^_between_ = 59.53%]. A statistically significant and medium-to-large effect size in increasing stereotypes toward people with disabilities was shown for simulation programs lasting for an hour or less (*d* = -.65, *SE* = .29, 95% *CI* [-1.21, -.08]). Subgroups of simulation programs lasting for more than 1 hour but within one day (*d* = .02, *SE* = .11, 95% *CI* [-.19, .23]), or 1–2 days (*d* = .06, *SE* = .23, 95% *CI* [-.38, .50]) showed small and statistically nonsignificant effect sizes in reducing the stereotypes.

Neither facilitation by people with disabilities nor the debriefing arrangement showed statistically significant associations with any between-subgroup differences in the program’s effectiveness in reducing stereotypes toward people with disabilities based on both the statistically nonsignificant *Q*_*B*_ and the relatively low *I*^2^_between_ statistics.

The posterior subgroup analysis showed statistically nonsignificant subgroup differences in the program’s effectiveness in reducing stereotypes toward people with disabilities between simulation programs using different referents in the measures of stereotypes toward people with disabilities [*Q*_*B*_(2) = 5.17, *p* = .08; *I*^2^_between_ = 61.28%] as well.

#### Publication bias assessment

Publication bias assessment was applied only to the pre-post comparison of stereotypes toward people with disabilities, as it was the only outcome comparison that fulfilled the convention of having at least 10 studies. Visual inspection of the funnel plots (S1 Fig) showed more or less even distributions of studies. Begg and Mazumdar’s tests reported statistically nonsignificant rank correlation (Kendall’s tau with continuity correction = 0.14, *z* = .62, *p* = .54). Egger’s test reported statistically nonsignificant intercept (Egger’s regression intercept = .13, *SE* = 1.12, 95% *CI* [-2.37, 2.63], *t*(10) = 0.12, *p* = .91). Duval and Tweedie’s [69] trim-and-fill method did not suggest any missing studies (reported number of studies trimmed = 0). No discrepancy between the observed effect size (point estimate = -.01) and adjusted effect size (point estimate = -.01) was observed. Results of these assessments overall did not suggest the presence of bias in the studies.

## Discussion

### Pre-to-post simulation comparisons

Significant changes in conceptions of social inclusion and emotions were observed at postsimulation that might represent relatively more direct and immediate reactions to the mobility disability simulation programs [31, 54]. First, the improvement in the conceptions of social inclusion at postsimulation shed light on a new perspective of the beneficial outcomes of these simulation programs. Second, a very large and statistically significant effect size in increasing anxiety, embarrassment, confusion, and helplessness at postsimulation was consistent with some past studies of simulation program effectiveness [8, 20, 27]. These emotional responses were in general regarded as undesired emotional change due to participation in disability simulation programs. This result thus empirically supported the suggested discontinuation of these disability simulation programs to avoid burden to participants and reinforcement of these “negative” and undesirable emotions toward people with disabilities. However, it should be noted that the small number of included studies and independent effect sizes for the pooling of effect sizes might hamper the validity of comparison and results interpretation. There were only two included studies with two corresponding independent effect sizes regarding the comparison on emotions before and after the participation in simulation program. There were only three included studies with five corresponding independent effect sizes regarding the comparison on the conceptions of social inclusion before and after the participation in simulation program. Cautions in results interpretation are needed.

It should also be noted that the use of scales such as the State-Trait Anxiety Inventory [71] in the studies reviewed were non-specific toward disability simulation context. It might hinder the empirical assessment of any contextualized emotional change due to participation in disability simulation programs. For example, taking the significant improvement in conception of social inclusion into account, the increase in anger and anxiety could plausibly be an indicator of an enhanced empathetic understanding of the psychological burden brought about by environmental inaccessibility for people with mobility disability [29–31]. Future empirical investigation to critically disentangle the mechanism, source, target, and the very nature of these emotional responses is highly warranted.

In practice, the large and significant increase in anger and anxiety upon simulation program completion must be handled appropriately at debriefing to minimize undesired psychological burden to participants and unintended reinforcement of ableist attitudes. It is recommended that, at debriefing, facilitators must encourage participants to first recognize and accept any emotional responses to the unfamiliar embodied experiences during the simulation program. For example, McGowan [31] stated that a post-simulation interview was conducted where the researcher helped the participants to explore, express, and articulate their strong emotional reactions to the simulation experiences. A brief practice of mindfulness-based stress reduction [72] might be conducted at debriefing to minimize lingering of the evoked intense and negative emotions, which might be followed by a spontaneous mutual sharing among the participants and facilitators where they might jointly decipher the actual sources and targets of their emotional reactions. Would these intensify the preexisting pity and fear toward disability? Would these emotional reactions become ambivalent emotions and/or righteous anger? Would these emotions target the injustice behind environmental inaccessibility encountered during the simulation programs, the simulated disability per se, or people with disabilities in society? If participants showed righteous anger toward the underlying injustice of environmental inaccessibility, the resulting changes might motivate the participants to engage in inclusion-affirming advocacy groups and collective actions for social inclusion based on the Social Identity Model of Collective Actions [29, 30, 73, 74]. Practical solutions to environmental inaccessibility might be coconstructed throughout the program process and especially during the debriefing session [3, 8, 9, 54].

As for the positive but inconclusive change in behavioral tendency at postsimulation, the particularly high heterogeneity within individual study effect sizes due to the variety in the inclusive behaviors measured (e.g., volunteer work, helping out research on environmental accessibility promotion, and performing various modifications of sports rules) in the included studies might obscure the pooled effect sizes. Other factors, such as the knowledge of the channels of advocacy actions for social inclusion and the perceived subjective norm of participation in advocacy, might be required in conjunction with the embodied simulation experiences to sufficiently motivate the tendency to engage in advocacy actions.

In addition, the pooled effect size in the change in stereotypes at postsimulation was nonsignificant and approached zero (i.e., neither increased nor decreased overall). The trend was basically consistent with that found by Flower and colleagues [6], showing very small effect sizes in improving attitudes (*d* = .04) by multiple types of disability simulation programs. Opposite mechanisms of attitude reevaluation in times of coping with embodied environmental inaccessibility might co-function as dual processes of stereotype change during the simulation programs. If there were a similar extent of the resulting positive and negative impacts on the reevaluation of ableist stereotypes, then the overall stereotype change might become very small or inconclusive. Further research on underlying mechanisms of any changes in stereotypes and behavioral tendency at postsimulation is warranted.

### Pre- to follow-up simulation comparisons

The large effects in reducing the stereotypes at follow-up but not at postsimulation might echo the suggested need of a certain period of time for newly constructed experiences during simulation programs to sufficiently integrate and manifest as reduced stereotypes [31, 54]. The intended impact of the simulation programs on stereotypes might appear later than the emotional change and the improved conception of social inclusion. While it might be common to readily experience anxiety under unfamiliar situations (i.e., simulating mobility disability), it might take a certain period of time to make sense of and assimilate these unfamiliar experiences. For instance, during the postsimulation period, participants are allowed more time and exposure to various daily life contexts to substantiate the comparison of their preexisting beliefs toward people with mobility disability and environmental in/accessibility from the perspectives of people without disability versus that from the perspectives of people with temporary mobility disability during the simulation programs. It takes time to construct new and less ableist beliefs of people with disabilities and environmental in/accessibility.

However, it is also noted that there were only three independent effect sizes from two included studies at follow-up assessment in the present meta-analysis. This result might hamper the validity of comparison and results interpretation. Further research on the detailed mechanisms of such possibly delayed stereotype change upon participation in simulation programs should be conducted. It is also recommended to incorporate follow-up assessments to keep track of participants’ longitudinal responses to obtain the wider scope of possible beneficial and detrimental consequences of simulation programs in addressing ableist attitudes more comprehensively. When follow-up assessments are not feasible, it is suggested to at least apply certain small-scale resources (e.g., leaflet and video showing real-life examples of debunking ableism) of linkage between the embodied simulation experiences, the emotions elicited, stereotype reduction, and behavioral advocacy for social inclusion as boosters during the postsimulation period to maximize reduction in ableist attitudes.

### Moderator testing results

The nonsignificant subgroup differences in the changes in stereotypes toward people with disabilities between pre-post simulation were inconsistent with the literature [3, 8, 9]. The number of independent studies within each subgroup might be insufficient to reveal any moderating effects of the three hypothesized categorical study-level characteristics through the subgroup analyses.

Despite the nonsignificant subgroup differences, a statistically significant and medium-to-large effect size in intensifying stereotypes toward people with disabilities by simulation programs lasting for an hour or less was found. It suggested that simulation programs involving mainly simulated mobility disability should last at least an hour or more to avoid undesirable increase in stereotypes toward people with disabilities. However, this result was inconsistent with that found by Flower and colleagues [6], showing positive effects in improving attitudes toward people with disabilities by multiple types of disability simulations that lasted 60 minutes or less. This finding revealed plausible insights brought about by a separate investigation of the effectiveness of each single or multiple types of simulation program to accumulate more empirical evidence for future cross-program comparison. Continual empirical comparisons of program effectiveness between different program durations in practice before further decisions on the optimal program duration are made is essential.

### Recommended reform in program design and evaluation

The reported program characteristics and methodological quality of the included studies in the present meta-analysis shed light on a series of recommended reform in the underlying rationales, along with the implementation and evaluation, rather than opting for actual, immediate program discontinuation of incorporating these programs in educational contexts at the current stage. Suggested reformed practices cover program message framing, formats of the simulation, scope and referents of outcome measures, incorporation of environmental perspectives and behavioral measures, and methodological quality of the program evaluation study.

#### Program message framing

The message framing of the relevant simulation programs should be critically reviewed, modified, and spelt out throughout the programs and reporting of the evaluation and findings. The findings showed that most of the empirical studies included in the present meta-analysis framed their simulation programs as “disability simulation”. Regarding the included simulation programs in the present meta-analysis, the core program content of most of the included simulation programs was indeed gaining embodied experiences of environmental in/accessibility.

Although it is undeniable that people living with mobility disability often experience environmental inaccessibility in everyday life, no simulation program can authentically capture the full scope of the multi-dimensional living experiences of people with disabilities [9, 15, 20, 32]. After all, no one could fully simulate the living experience of one another, regardless of the disability status. Instead of focusing on “simulating people with disabilities” by applying certain assistive devices and/or external modifications to the participants (e.g. try sitting on wheelchairs for a period of time), it is recommended to position the core program aims by orienting the program framing and participants toward paying attention to the causes, manifestations, and practical solutions of environmental inaccessibility to challenge ableism.

In addition, it is recommended to elaborate the concepts of “disability” at the briefing and debriefing sessions is essential to align participants’ relevant concepts. Disability indeed can be conceptualized by a number of models of disability such as the medical, charity, social, and human rights model of disability [75] that cover multifarious domains of construction and expression of “disability”. It is suggested to introduce the social and human rights models of disability to the participants to let them understand the social-environmental construction of disability experience instead of the medicalized orientation of disability. Otherwise, some participants might resort to mistakenly induce the simple but deeply-anchored association of the barriers encountered in the simulation programs and the temporarily simulated identity of people with mobility disability, reinforcing a stereotypically unidimensional and deficit-oriented conceptualization of “disability”.

#### Formats of mobility disability simulation

The variations in the execution of different formats of mobility disability simulation should be carefully considered and reported in greater detail. It is also recommended to assess and report the levels of stigma toward the assistive devices (e.g., wheelchairs and crutches) used to simulate mobility disability as one of the baseline characteristics and/or outcome variable in future studies. Stigma attached to the use of different assistive devices and the anticipated program outcomes (e.g., attitudes toward people with mobility disability and in/accessibility) might be associated [76–79].

The most common format of mobility disability simulation among the empirical studies included in the present meta-analysis was wheelchair use. Most of the empirical studies included in the present meta-analysis did not describe clearly whether the participants independently maneuvered the wheelchairs themselves, or their wheelchairs were passively propelled by other participants, or both, during the simulation programs. The participants involved were principally people without disability and not wheelchair users as well. However, this mere difference might affect the interpretation of study findings.

Galli and colleagues [77, 78] found that expert users of wheelchairs (such as some people with mobility disability) and nonexpert users whose wheelchairs were propelled by others (like those participants of the simulation programs examined in the present meta-analysis), but not novice users who propelled the wheelchair themselves, showed an extended peripersonal space and enhanced body-environment interactions through simultaneously integrating information from their own body and the external environment in which their body was acting. Body-environment interactions might facilitate embodied experiences as well as the appraisal and manifestations of the experiences. The enhancement effects were not shown among nonexpert users who actively maneuvered their own wheelchairs, which might be caused by focusing attention on the physical effort in maneuvering the wheels themselves. Therefore, the use of any assistive devices during simulation programs might influence participants’ observations, interpretations, and interactions with any environmental in/accessibility in the surrounding environment during the simulation programs, thereby influencing the anticipated program effects.

#### Alternative format of disability simulation

Given the possible pitfalls of the message framing and execution format of mobility disability simulation reviewed among the studies included in the present meta-analysis, the program format might be modified to orient participants toward the manifestations and underlying causes of environmental in/accessibility to challenge ableism. An alternative format of disability simulation is suggested.

Participants without disability might be invited to walk around the community “as usual”, without the need to use any assistive devices such as wheelchairs to “simulate” people with mobility disability. However, during the simulation programs, participants are still expected to encounter certain in/accessibility experiences. For instance, they are not allowed to get on transportation whenever the International Symbol of Access (which features the image of a wheelchair user) is shown on that public transportation such as a bus [80]. This suggested simulation format does not ask participants to physically simulate the life of another group of persons by using certain assistive devices or intentionally performing some tasks, but to focus on each of participant’s own life and the environmental context as encountered.

The message to be conveyed by this alternative format is that while participants without disability are traditionally regarded as the “*able-bodied*” and “*normal*” groups under ableist social systems, they would readily become “*disabled*” and “*abnormal*” due to environmental inaccessibility when exclusive social functioning and environmental design do not take the inherent normality of differences among individuals into account at the outset. This experience could dismantle the seemingly clear boundary between “*normal*” versus “*abnormal*” and between “*abled*” versus “*disabled*” that are associated with the deeply rooted ableism. It might then help establish the shared reality of why a non-ableist society is essential and how a non-ableist society should manifest for different individuals, regardless of their disability status, to resume spatial justice and to match the universal design movement. It might also challenge the stereotypes that the “impairment status” of people with certain disabilities is the core cause of the “inevitable” barriers they encounter in everyday life. This modified program format might better match the ultimate purpose of simulation programs, which is to orient participants toward the manifestations, causes, and practical solutions of environmental inaccessibility to challenge ableism.

#### Expanded scope of outcome assessments

It is suggested that qualitative data of the outcome variables could be collected before and after the simulation through open-ended questions or interviews. It would supplement the interpretation of the quantitative data collected through self-report surveys.

#### Referents of the outcome measures

The referents of the measures of stereotypes were not uniform across all the empirical studies included in the meta-analysis. These studies adopted the referents of mainly “people with disabilities”, “people with physical disability”, or “disabled persons” without clearly defining the referents. Subgroup analysis in the present meta-analysis showed that there were no statistically significant differences in program effectiveness in reducing the stereotypes between those programs using referents of “people with disabilities”, “people with physical disability”, or “disabled persons” in the present meta-analysis. However, these referents could conceptually cover people with a wide variety and combination of disability statuses of living experiences, social and environmental barriers [81].

The Baseline Survey on Public Attitudes toward Persons with a Disability conducted by the Equal Opportunities Commission [82] of Hong Kong found that, without prompting, most of the respondents said that “people with disabilities” conceptually referred to “people with mobility disability” (93%) or “people with sensory impairment” (74%). However, the referent “people with disabilities” should conceptually cover a much wider scope of disability experiences, such as people with mental illness and people with chronic illness. Without clearly defining the terminology of these disability-related referents in the simulation programs and outcome measures, it is plausible that the interpretations of these referents by different stakeholders such as the researchers, program facilitators, participants, and readers of the research reports might be inconsistent from the program design to the actual implementation. Past studies have also established a hierarchy of attitudes and acceptance toward different disability groups [83–85]. Therefore, the inconsistency of the referents along with the lack of clear definitions might further inhibit the validity of the outcome assessments and results interpretations. The choice of referents should be carefully considered and explicitly defined in each simulation program and evaluation study.

#### Incorporation of environmental perspectives

A lack of empirical assessment of the changes in the conception of environmental in/accessibility was observed in the included studies in the present meta-analysis. The Attitudes Toward Disabled Persons (Forms A/B/O) Scales [70] was the most frequently employed instrument measuring ableist social attitudes in the studies included in the present meta-analysis. The Attitudes Toward Disabled Persons (Forms A/B/O) Scales has been the classic and principal outcome measure of attitudes toward people with disabilities as an assessment of the effectiveness of disability-related simulation programs. Nevertheless, the use of the Attitudes Toward Disabled Persons (Forms A/B/O) Scales has been questioned for its unidimensional (i.e., mainly cognitive) and negatively framed items as well as the incompatibility with the essential up-to-date recognized ecological approach to ableism [86]. Simulation programs under the present examination heavily involve person-environmental interactions. Results of the present meta-analysis calls for the development of measurement tools that can assess the multidimensions of attitudes toward people with disabilities and the conception of environmental in/accessibility [3, 31].

#### Behavioral measures

A lack of outcome assessment and reported data of discriminatory behaviors against people with disabilities was also found in the included studies in the present meta-analysis. Assessment tools of the actual behaviors for promoting social inclusion and advocacy engagement should be developed for use in future evaluation of these simulation programs.

#### Methodological quality of the simulation program evaluation study

The methodological quality of the included studies was not satisfactory. Eight out of twelve studies employed before-and-after comparisons, among which, five were uncontrolled studies without follow-up assessments. Of the twelve included studies, only one study employed randomized controlled trials with follow-up assessments. Randomized controlled trials, but not before-and-after studies, should be relatively the most valid experimental design to delineate the causality of simulation programs on any changes in ableist attitudinal outcomes controlling for other factors. Moreover, six of the seven controlled studies did not clearly report any significant differences in demographics and outcome variables between the comparison groups at baseline. Only two included studies included follow-up assessments, which limited the examination of any sustained impact on modifying the ableist social attitudes. The lack of methodological rigor in various domains of the program design and evaluations might seriously hinder the outcome assessment and results interpretation. It is highly recommended to employ more rigorous randomized controlled trials with follow-up assessments of the program effectiveness in addressing ableist attitudes.

### Limitations of the present review

There were several limitations of the present meta-analysis. First, the number of included studies with the corresponding independent effect sizes and sample sizes for the pooling of effect sizes, heterogeneity assessment, and subgroup analysis, was relatively small. It might hamper the validity of the pooling of the effect sizes, effects comparisons and results interpretation. Cautions in results interpretation are needed. A lack of representation of Asian countries and cities in the coverage of the geographical origin of the included studies was observed. The sample types of the 12 included articles were not homogeneous. Among the 12 included articles, seven articles involved undergraduates and postgraduates. Four articles involved participants of children aged below 18. One article involved community adults as the participants. Among the seven articles involving undergraduates and postgraduates, there was also a variety in the academic study program of these students, such as physiotherapy, psychology, and physical education training from public educational centers. Given the variety in the sample types and the relatively small number of included studies for valid and meaningful subgroup analyses, moderator testing on the sample type was not conducted. In addition, five out of the 12 included articles reported whether the participants had any prior experience of contact with people with disability; but empirical data of the level of contact experience and knowledge of the participants toward people with disability at baseline was unavailable for further moderator testing. Furthermore, empirical data for the follow-up assessments were particularly lacking. A lack of outcome assessment and reported data of discriminatory behaviors against people with disability and the conception of environmental in/accessibility was also found in the included studies in the present meta-analysis. The methodological quality of the included studies was not satisfactory.

Second, the citation search was limited to publications written in English, although the reference list of a published systematic review on disability awareness programs [56] that was written in Spanish was included in the citation search to identify studies that might be missed in the online database search. Third, the studies included in the present meta-analysis focused on the simulation of environmental in/accessibility in the presence of simulated mobility disability. The findings might not be generalized to all kinds of programs of simulation of environmental in/accessibility in the presence of simulation of other types of disability, such as the associations of Deaf Space and the Deaf community and hard-of-hearing individuals [87]. Experiences of having mixed types of disabilities should be further considered as well. Finally, subgroup comparisons were only observational, and no causal inference of any of the study-level characteristics on any observed between-group differences in the summary effect sizes could be drawn [65].

## Conclusions

The present meta-analysis quantitatively reviewed 12 empirical studies of the effectiveness of simulation of environmental in/accessibility in the presence of simulated mobility disability in addressing ableist attitudes toward people with disabilities and conception of social inclusion in terms of stereotypes, emotions, and behaviors. Opposing and inconclusive results were found. The findings also inform a series of recommended reforms in the rationale of the design, implementation, and evaluation of these simulation programs. Recommended practice reform covers program message framing, formats of the simulation, scope and referents of outcome measures, incorporation of environmental perspectives and behavioral measures, and methodological quality of the program evaluation study. It is suggested to first carry out the recommended practice reform before any further concrete, black-and-white decisions on the suggested program discontinuation are to be made in the future. The findings of the present meta-analysis enrich the current research on ableism and disability simulation programs to inform program practice and the use of these programs across educational contexts.

## Supporting information

S1 Table PRISMA 2009 checklist. (PDF)

S2 Table The 22 adapted methodological quality assessment criteria. (PDF)

S3 Table Study characteristics, sample characteristics, and relevant outcomes of the included studies. SIM = simulation group; CON = control group.
(PDF)

S4 Table Summary of the main characteristics of the studies included for meta-analysis. The study characteristics are categorized and summarized by the authors. Please refer to the S3 Table and the full-texts of the original studies for detailed information. Categories were arranged in descending order of the number of articles except the year of publication.
(PDF)

S5 Table Results of the methodological quality assessment. + indicated criteria fulfilled;—indicated criteria not fulfilled; CD = cannot determined based on the reported information; NA = not applicable; NR = information not reported. Criteria of the methodological quality assessment were listed in the S2 Table.
(PDF)

S1 Fig Funnel plot for the pre-post effect on modifying stereotype. (TIFF)

S1 File The electronic search strategy for Web of Science. (PDF)

S2 File Dataset for effect size calculations. (CMA)

S3 File References of the 12 studies included in the present meta-analysis. (PDF)
