# Supplementary material for: Meta-analysis of studies on the impact of mobility disability simulation programs on attitudes toward people with disabilities and environmental in/accessibility
Source: PLoS One. 2022 Jun 10;17(6):e0269357. doi: 10.1371/journal.pone.0269357 (PMC9187118; doi:10.1371/journal.pone.0269357)
Supplement: S2 Table — (PDF) [file pone.0269357.s002.pdf]

S2 Table. The 22 adapted methodological quality assessment criteria.

Item	Assessment criteria
QA1	Was the study question or objective clearly stated?
QA2	Were eligibility/selection criteria for the study population prespecified and clearly described?
QA3	Were all eligible participants that met the prespecified entry criteria enrolled?
QA4	Were the participants in the study representative of those who would be eligible for the test/service/intervention in the general or clinical population of interest?
QA5	Was the study described as randomized, a randomized trial, a randomized clinical trial, or a randomized controlled trial?
QA6	Was the method of randomization adequate (i.e., use of randomly generated assignment)?
QA7	Was the treatment allocation concealed (so that assignments could not be predicted)?
QA8	Were study participants blinded to treatment group assignment?
QA9	Were study providers blinded to treatment group assignment?
QA10	Were the people assessing the outcomes blinded to the participants' group assignments?
QA11	Were the groups similar at baseline on important characteristics that could affect outcomes (e.g., demographics, risk factors, co-morbid conditions)?
QA12	Was the overall drop-out rate from the study at endpoint 20% or lower of the number allocated to treatment (or those participated in before-and-after study)?
QA13	Was the test/service/intervention clearly described and delivered consistently across the study population?
QA14	Was the differential drop-out rate (between treatment groups) at endpoint 15 percentage points or lower?
QA15	Was there high adherence to the intervention protocols for each treatment group?
QA16	Were other interventions avoided or similar in the groups (e.g., similar background treatments)?
QA17	Were outcomes assessed using valid and reliable measures, implemented consistently across all study participants?
QA18	Did the authors report that the sample size was sufficiently large to be able to detect a difference in the main outcome between groups with at least 80% power?
QA19	Were outcome measures of interest taken multiple times before the intervention and multiple times after the intervention (i.e., did they use an interrupted time-series design)?

QA20	Were outcomes reported or subgroups analyzed prespecified (i.e., identified before analyses were conducted)?
QA21	Were all analyzed in the group to which they were originally assigned, i.e., did they use an intention-to-treat analysis?
QA22	Did the statistical methods examine changes in outcome measures from before to after the intervention? Were statistical tests done that provided p-values for the pre-to-post changes; or the 95% confidence intervals for the estimates of change?

References:

National Heart Lung and Blood Institute. Quality Assessment of Controlled Intervention Studies 2014 [Available from: https://www.nhlbi.nih.gov/health-topics/study-quality-assessment-tools]

National Heart Lung and Blood Institute. Quality Assessment Tool for Before-After (Pre-Post) Studies With No Control Group 2014 [Available from: https://www.nhlbi.nih.gov/health-topics/study-quality-assessment-tools]
