# Supplementary material for: Meta-analysis of studies on the impact of mobility disability simulation programs on attitudes toward people with disabilities and environmental in/accessibility
Source: PLoS One. 2022 Jun 10;17(6):e0269357. doi: 10.1371/journal.pone.0269357 (PMC9187118; doi:10.1371/journal.pone.0269357)
Supplement: S3 Table — SIM = simulation group; CON = control group. (PDF) [file pone.0269357.s003.pdf]

S3 Table. Study characteristics, sample characteristics, and relevant outcomes of the included studies.

Study (Arranged in ascending order of publication year)	Location (First author)	Participants				Embodied simulation program design					
		Location	Sample size	Sample type	Mean age (years)	Format of simulating mobility disability	Settings	Program duration	Deliverers	Ableist attitudes-related outcomes	Instruments
Semple et al., 1980	Canada	Canada	SIM = 18<br>CON = 14	Physiotherapy students	19.44	Wheelchair only	Not reported	1-2 days	Course instructors	Stereotypes toward people with disability	Attitudes Toward Disabled Persons – Form A (Yuker & Block, 1986)
Vargo et al., 1981	USA	USA	A) SIM = 15<br>CON = 9<br>B) SIM = 18<br>CON1 = 10<br>CON2 = 22	Physiotherapy students	A) 19.33<br>B) SIM = 19.44<br>CON = 19.82	Wheelchair only	Community	1-2 days	Course instructors	Stereotypes toward people with disability	Attitudes Toward Disabled Persons – Form A (Yuker & Block, 1986)
Avery & Davis, 1983	USA	USA	SIM = 16<br>CON = 16	Preservice vocational teachers	Range = 18-26	Prosthetic limbs only	Vocational evaluation laboratory at university	1 hour 40 mins	Copyrighted standardized film strip sound cassette (audio-visual) presentation	Stereotypes toward people with disability	Attitudes Toward Disabled Persons – Form O (Yuker & Block, 1986)
										Overall emotional changes	State-Trait Anxiety Inventory (Spielberger, 1970)
Houston, 1991	USA	USA	33	Persons 18 years or older	Not reported	Wheelchair only	Civic center / shopping mall	Not reported	Researchers	Stereotypes toward people with disability	Attitudes Toward Disabled Persons (Yuker & Block, 1986)

		Participants				Embodied simulation program design					
**Study (Arranged in ascending order of publication year)**	**Location (First author)**	**Location**	**Sample size**	**Sample type**	**Mean age (years)**	**Format of simulating mobility disability**	**Settings**	**Program duration**	**Deliverers**	**Ableist attitudes-related outcomes**	**Instruments**
Grayson & Marini, 1996	USA	USA	SIM = 20<br>CON = 18	Graduate students	Not reported	Wheelchair only	Not reported	Not reported	Instructor	Stereotypes toward people with disability	Self-constructed
McGowan, 1998	USA	USA	SIM = 20<br>CON = 20	Undergraduates in Psychology	Not reported	Wheelchair or crutches	Campus	60 mins	Researchers	Stereotypes toward people with disability	Attitudes Toward Disabled Persons – Form A (Yuker & Block, 1986)
										Behavioral tendency of inclusion-promoting actions	Disability Interest Questionnaire (Clore & Jeffery, 1972)
Xafopoulos et al., 2009	Belgium	Czech Republic	Boys = 29<br>Girls = 42	Students	Boys = 11.67<br>Girls = 12.7	sledge hockey, wheelchair, wheelchair basketball, boccia	School	40 mins each for 6 activities	Researchers	Stereotypes toward people with disabilities	The Adjective Checklist (Siperstein, 1980)
										Conception of social inclusion	Children's Attitude Toward Integrated Physical Education-Revised (Block, 1995)
										Behavioral tendency of inclusion-promoting actions	
Liu et al., 2010	China	Czech Republic	Boys = 17<br>Girls = 19	Grade 6 elementary school children	Boys = 11.88<br>Girls = 11.68	Wheelchair basketball	School	15 mins	School teachers and athletes with disability	Stereotypes toward people with disabilities	The Adjective Checklist (Siperstein, 1980)

Study (Arranged in ascending order of publication year)	Location (First author)	Participants				Embodied simulation program design					
		**Location**	**Sample size**	**Sample type**	**Mean age (years)**	**Format of simulating mobility disability**	**Settings**	**Program duration**	**Deliverers**	**Ableist attitudes-related outcomes**	**Instruments**
										Conception of social inclusion<br>Behavioral tendency of inclusion-promoting actions	Children's Attitude Toward Integrated Physical Education-Revised (Block, 1995)
Papaioannou et al., 2013	Greece	Greece	SIM = 197<br>CON = 190	Children	SIM = 13.08<br>CON = 13.43	Wheelchair basketball	Campsite	35 mins for each of the 10 activities	Researchers and trained undergraduates	Conception of social inclusion<br>Behavioral tendency of inclusion-promoting actions	Children's Attitude Toward Integrated Physical Education-Revised (Block, 1995)
Nario-Redmond et al., 2017	USA	USA	60	College students	20	Wheelchair only	Not reported	Not reported	Not reported	Overall emotional changes	Profile of Mood States-Short Form (EdITS, 1999)
Silverman et al., 2018	USA	USA	SIM = 16<br>CON = 16	First-year Master of Occupational Therapy and Doctor of Physical Therapy students	SIM = 27.00<br>CON = 26.88	Wheelchair and other assistive devices	School	Not reported	Physical therapist	Stereotypes toward people with disabilities	Self-constructed
Reina et al., 2021	Spain	Spain	241	Physical Education students from public educational centers	13.14 (overall sample)	A combination of format	School	45-50 mins each session	Trained Physical Education teachers	Behavioral tendency of inclusion-promoting actions	Attitudes Towards Inclusion in PE Questionnaire (ATIPEQ)

SIM = simulation group; CON = control group.

References of the 12 studies included in the present meta-analysis:

1. Semple JE, Vargo JW, Vargo FA. Disability simulation and its effect on changing the attitudes of physical therapy students towards disabled persons: Some preliminary experimental results. N Z J Physiother. 1980;8(2): 6-8.
2. Vargo JW, Vargo FA, Semple JE. The influence of disability simulation on the attitudes of physical therapy students toward disabled persons. N Z J Physiother. 1981;9: 22-25.
3. Avery MR, Davis PD. The effect of anxiety producing simulation tasks on nondisabled preparatory vocational teachers' attitudes toward the physically handicapped vocational student. J Vocat Educ Res. 1983;8(4): 1-10.
4. Houston L. The effect of task difficulty level in disability simulation [Ph.D. dissertation]: Oklahoma State University; 1991.
5. Grayson E, Marini I. Simulated disability exercises and their impact on attitudes toward persons with disabilities. Int J Rehabil Res. 1996;19(2): 123-131.
6. McGowan JP. The effects of disability simulations on attitudes toward persons with disabilities [Ph.D. dissertation]. New Jersey, United States: Seton Hall University; 1998.
7. Xafopoulos G, Kudláček M, Evaggelinou C. Effect of the intervention program "Paralympic School Day" on attitudes of children attending international school towards inclusion of students with disabilities. Acta Gymnica. 2009;39(4): 63-71.
8. Liu Y, Kudláček M, Ješina O. The influence of Paralympic School Day on children's attitudes towards people with disabilities. Acta Gymnica. 2010;40(2): 63-69.
9. Papaioannou C, Evaggelinou C, Barkoukis V, Block ME. Disability awareness program in a summer camp. Eur J Adapt Phys Act. 2013;6(3): 19-28.
10. Nario-Redmond MR, Gospodinov D, Cobb A. Crip for a day: The unintended negative consequences of disability simulations. Rehabil Psychol. 2017;62(3): 324-333.
11. Silverman AM, Pitonyak JS, Nelson IK, Matsuda PN, Kartin D, Molton IR. Instilling positive beliefs about disabilities: Pilot testing a novel experiential learning activity for rehabilitation students. Disabil Rehabil. 2018;40(9): 1108-1113.
12. Reina R, Haegele JA, Pérez-Torralba A, Carbonell-Hernández L, Roldan A. The influence of a teacher-designed and -implemented disability awareness programme on the attitudes of students toward inclusion. Eur Phys Educ Rev. 2021.

Other references:

1. Yuker HE, Block JR. Research with the Attitude Toward Disabled Persons scales (ATDP) 1960-1985: Hofstra University; 1986.
2. Speilberger C, Gorsuch R, Lushene R. STAI manual. Palo Alto, CA: Consulting Psychologists Press, Inc. 1970.
3. Clore GL, Jeffery KM. Emotional role playing, attitude change, and attraction toward a disabled person. J Pers Soc Psychol. 1972;23: 105-111.
4. Siperstein, GN. Instruments for measuring children's attitudes toward the handicapped (Unpublished manuscript). Boston: University of Massachusetts. 1980.
5. Block MB. Development and validation of Children's Attitudes Toward Integrated Physical Education-Revised (CAIPE-R) Inventory. Adapt Phys Activ Q. 1995;12: 60-77.
6. EdITS. EdITS research and developments. San Diego, CA: EdITS. 1999.
