# Supplementary material for: Meta-analysis of studies on the impact of mobility disability simulation programs on attitudes toward people with disabilities and environmental in/accessibility
Source: PLoS One. 2022 Jun 10;17(6):e0269357. doi: 10.1371/journal.pone.0269357 (PMC9187118; doi:10.1371/journal.pone.0269357)
Supplement: S4 Table — The study characteristics are categorized and summarized by the authors. Please refer to the S3 Table and the full-texts of the original studies for detailed information. Categories were arranged in descending order of the number of articles except the year of publication. (PDF) [file pone.0269357.s004.pdf]

S4 Table. Summary of the main characteristics of the studies included for meta-analysis.

	Number of articles
Country of origin of the participants	
USA	7
Czech Republic	2
Canada	1
Greece	1
Spain	1
Sample type	
Undergraduates and postgraduates	7
Children aged below 18	4
Community adults	1
Format of simulating mobility disability	
Using wheelchairs only	7
Using combinations of assistive devices, e.g., wheelchairs and crutches	3
Using prosthetic limb only	1
A combination of format	1
Settings	
School campus	5
Community	4
Not reported	3
Outcomes related ableist cognitions / emotions / behaviors	
Stereotypes toward people with disabilities	9
Behavioral tendency of inclusion-promoting actions	5
Conception of social inclusion	3
Overall emotional changes	2
Instruments	
Attitudes Toward Disabled Persons (Forms A/B/O) Scales (Yuker & Block, 1986)	5
Children's Attitude Toward Integrated Physical Education-Revised (Block, 1995)	3
Self-constructed	2
The Adjective Checklist (Siperstein, 1980)	1
Disability Interest Questionnaire (Clore & Jeffery, 1972)	1

Profile of Mood States-Short Form (EdITS, 1999)	1
State-Trait Anxiety Inventory (Spielberger, 1970)	1
Attitudes Towards Inclusion in PE Questionnaire (ATIPEQ)	1
Year of publication	
1980-1990	3
1991-2000	3
2001-2010	2
2011-2021	4
Publication type	
Journal articles	10
Unpublished doctoral dissertations	2

The study characteristics are categorized and summarized by the authors. Please refer to the S3 Table and the full-texts of the original studies for detailed information. Categories were arranged in descending order of the number of articles except the year of publication.

References:

1. Yuker HE, Block JR. Research with the Attitude Toward Disabled Persons scales (ATDP) 1960-1985: Hofstra University; 1986.
2. Block MB. Development and validation of Children's Attitudes Toward Integrated Physical Education-Revised (CAIPE-R) Inventory. Adapt Phys Activ Q. 1995;12: 60-77.
3. Siperstein, GN. Instruments for measuring children's attitudes toward the handicapped (Unpublished manuscript). Boston: University of Massachusetts. 1980.
4. Clore GL, Jeffery KM. Emotional role playing, attitude change, and attraction toward a disabled person. J Pers Soc Psychol. 1972;23: 105-111.
5. EdITS. EdITS research and developments. San Diego, CA: EdITS. 1999.
6. Speilberger C, Gorsuch R, Lushene R. STAI manual. Palo Alto, CA: Consulting Psychologists Press, Inc. 1970.
