# Supplementary material for: Meta-analysis of studies on the impact of mobility disability simulation programs on attitudes toward people with disabilities and environmental in/accessibility
Source: PLoS One. 2022 Jun 10;17(6):e0269357. doi: 10.1371/journal.pone.0269357 (PMC9187118; doi:10.1371/journal.pone.0269357)
Supplement: S5 Table — + indicated criteria fulfilled;—indicated criteria not fulfilled; CD = cannot determined based on the reported information; NA = not applicable; NR = information not reported. Criteria of the methodological quality assessment were listed in the S2 Table. (PDF) [file pone.0269357.s005.pdf]

S5 Table. Results of the methodological quality assessment.

Study	QA1	QA2	QA3	QA4	QA5	QA6	QA7	QA8	QA9	QA10	QA11	QA12	QA13	QA14	QA15	QA16	QA17	QA18	QA19	QA20	QA21	QA22
Semple et al., 1980	+	NR	NR	-	NA	NA	-	+	-	-	NR	+	+	+	NR	NR	+	NR	+	+	-	+
Vargo et al., 1981	+	NR	NR	-	NA	NA	-	+	-	-	NR	+	+	+	NR	NR	+	NR	+	+	NR	+
Avery & Davis, 1983	+	NR	NR	+	+	+	CD	+	-	CD	NR	+	+	NR	+	+	+	-	+	+	NR	+
Houston, 1991	+	+	+	-	NA	NA	NA	NR	-	NR	NA	NR	+	NR	+	+	+	NR	-	+	-	+
Grayson & Marini, 1996	+	NR	NR	-	-	NA	CD	NR	-	NR	NR	+	+	+	NR	NR	CD	NR	-	+	+	+
McGowan, 1998	+	NR	NR	-	+	-	+	NR	-	NR	NR	NR	+	+	+	+	+	NR	-	+	NR	NA
Xafopoulos et al., 2009	+	NR	NR	-	NA	NA	NA	NR	-	NR	NA	NR	+	NR	NR	+	+	NR	-	+	NR	+
Liu et al., 2010	+	NR	NR	-	NA	NA	NA	NR	-	NR	NA	NR	+	NR	NR	+	+	NR	-	+	NR	-
Papaioannou et al., 2013	+	NR	NR	-	NA	NA	NR	NA	-	CD	NR	NR	+	NR	+	NR	+	NR	-	+	NR	+
Nario-Redmond et al., 2017	+	NR	NR	-	-	NA	NA	NR	NR	NR	NA	+	+	+	NR	+	+	NR	-	+	CD	+
Silverman et al., 2018	+	NR	NR	-	+	+	+	+	-	NR	+	+	+	+	+	+	CD	NR	-	+	NR	+
Reina et al., 2021	+	NR	NR	-	-	NA	NA	NR	-	-	NA	+	NR	+	NA	+	+	NR	-	+	NA	+

+ indicated criteria fulfilled; - indicated criteria not fulfilled; CD = cannot determined based on the reported information; NA = not applicable; NR = information not reported. Criteria of the methodological quality assessment were listed in the S2 Table.

References of the 12 studies included in the present meta-analysis:

1. Semple JE, Vargo JW, Vargo FA. Disability simulation and its effect on changing the attitudes of physical therapy students towards disabled persons: Some preliminary experimental results. N Z J Physiother. 1980;8(2): 6-8.
2. Vargo JW, Vargo FA, Semple JE. The influence of disability simulation on the attitudes of physical therapy students toward disabled persons. N Z J Physiother. 1981;9: 22-25.

3. Avery MR, Davis PD. The effect of anxiety producing simulation tasks on nondisabled preparatory vocational teachers' attitudes toward the physically handicapped vocational student. J Vocat Educ Res. 1983;8(4): 1-10.
4. Houston L. The effect of task difficulty level in disability simulation [Ph.D. dissertation]: Oklahoma State University; 1991.
5. Grayson E, Marini I. Simulated disability exercises and their impact on attitudes toward persons with disabilities. Int J Rehabil Res. 1996;19(2): 123-131.
6. McGowan JP. The effects of disability simulations on attitudes toward persons with disabilities [Ph.D. dissertation]. New Jersey, United States: Seton Hall University; 1998.
7. Xafopoulos G, Kudláček M, Evaggelinou C. Effect of the intervention program “Paralympic School Day” on attitudes of children attending international school towards inclusion of students with disabilities. Acta Gymnica. 2009;39(4): 63-71.
8. Liu Y, Kudláček M, Ješina O. The influence of Paralympic School Day on children's attitudes towards people with disabilities. Acta Gymnica. 2010;40(2): 63-69.
9. Papaioannou C, Evaggelinou C, Barkoukis V, Block ME. Disability awareness program in a summer camp. Eur J Adapt Phys Act. 2013;6(3): 19-28.
10. Nario-Redmond MR, Gospodinov D, Cobb A. Crip for a day: The unintended negative consequences of disability simulations. Rehabil Psychol. 2017;62(3): 324-333.
11. Silverman AM, Pitonyak JS, Nelson IK, Matsuda PN, Kartin D, Molton IR. Instilling positive beliefs about disabilities: Pilot testing a novel experiential learning activity for rehabilitation students. Disabil Rehabil. 2018;40(9): 1108-1113.
12. Reina R, Haegele JA, Pérez-Torralba A, Carbonell-Hernández L, Roldan A. The influence of a teacher-designed and -implemented disability awareness programme on the attitudes of students toward inclusion. Eur Phys Educ Rev. 2021.
