# Supplementary figures and images for: Meta-analysis of studies on the impact of mobility disability simulation programs on attitudes toward people with disabilities and environmental in/accessibility
Source: PLoS One. 2022 Jun 10;17(6):e0269357. doi: 10.1371/journal.pone.0269357 (PMC9187118; doi:10.1371/journal.pone.0269357)

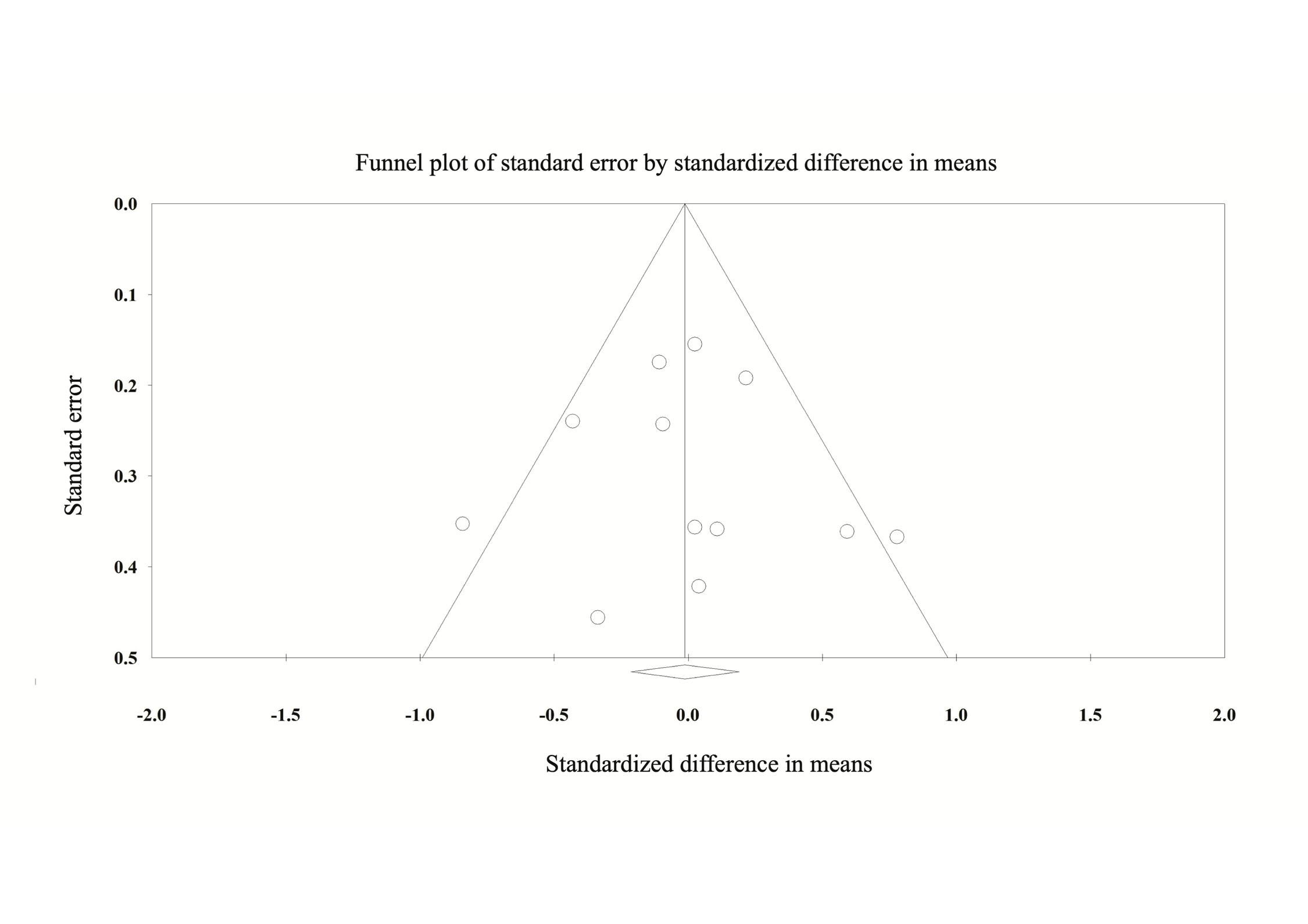

Supplement: S1 Fig — (TIFF) [file pone.0269357.s006.tiff]
