# Supplementary material for: Meta-analysis of studies on the impact of mobility disability simulation programs on attitudes toward people with disabilities and environmental in/accessibility
Source: PLoS One. 2022 Jun 10;17(6):e0269357. doi: 10.1371/journal.pone.0269357 (PMC9187118; doi:10.1371/journal.pone.0269357)
Supplement: S1 File — (PDF) [file pone.0269357.s007.pdf]

S1 File. The electronic search strategy for Web of Science.

(TI=(disability OR disabilities OR disabled OR handicap OR handicaps OR handicapped OR handicapping OR impairment OR impairments OR impaired) AND TI=(experiential OR simulation OR simulations OR simulated OR simulated OR awareness)) AND LANGUAGE: (English)
