# Supplementary material for: Meta-analysis of studies on the impact of mobility disability simulation programs on attitudes toward people with disabilities and environmental in/accessibility
Source: PLoS One. 2022 Jun 10;17(6):e0269357. doi: 10.1371/journal.pone.0269357 (PMC9187118; doi:10.1371/journal.pone.0269357)
Supplement: S3 File — (PDF) [file pone.0269357.s009.pdf]

S3 File. References of the 12 studies included in the present meta-analysis.

1. Semple JE, Vargo JW, Vargo FA. Disability simulation and its effect on changing the attitudes of physical therapy students towards disabled persons: Some preliminary experimental results. N Z J Physiother. 1980;8(2): 6-8.
2. Vargo JW, Vargo FA, Semple JE. The influence of disability simulation on the attitudes of physical therapy students toward disabled persons. N Z J Physiother. 1981;9: 22-25.
3. Avery MR, Davis PD. The effect of anxiety producing simulation tasks on nondisabled preparatory vocational teachers' attitudes toward the physically handicapped vocational student. J Vocat Educ Res. 1983;8(4): 1-10.
4. Houston L. The effect of task difficulty level in disability simulation [Ph.D. dissertation]: Oklahoma State University; 1991.
5. Grayson E, Marini I. Simulated disability exercises and their impact on attitudes toward persons with disabilities. Int J Rehabil Res. 1996;19(2): 123-131.
6. McGowan JP. The effects of disability simulations on attitudes toward persons with disabilities [Ph.D. dissertation]. New Jersey, United States: Seton Hall University; 1998.
7. Xafopoulos G, Kudláček M, Evaggelinou C. Effect of the intervention program "Paralympic School Day" on attitudes of children attending international school towards inclusion of students with disabilities. Acta Gymnica. 2009;39(4): 63-71.
8. Liu Y, Kudláček M, Ješina O. The influence of Paralympic School Day on children's attitudes towards people with disabilities. Acta Gymnica. 2010;40(2): 63-69.
9. Papaioannou C, Evaggelinou C, Barkoukis V, Block ME. Disability awareness program in a summer camp. Eur J Adapt Phys Act. 2013;6(3): 19-28.
10. Nario-Redmond MR, Gospodinov D, Cobb A. Crip for a day: The unintended negative consequences of disability simulations. Rehabil Psychol. 2017;62(3): 324-333.
11. Silverman AM, Pitonyak JS, Nelson IK, Matsuda PN, Kartin D, Molton IR. Instilling positive beliefs about disabilities: Pilot testing a novel experiential learning activity for rehabilitation students. Disabil Rehabil. 2018;40(9): 1108-1113.
12. Reina R, Haegele JA, Pérez-Torralba A, Carbonell-Hernández L, Roldan A. The influence of a teacher-designed and -implemented disability awareness programme on the attitudes of students toward inclusion. Eur Phys Educ Rev. 2021.
